# Dysregulation of Amino Acid, Lipid, and Acylpyruvate Metabolism in Idiopathic Intracranial Hypertension: A Non-targeted Case Control and Longitudinal Metabolomic Study

**DOI:** 10.1021/acs.jproteome.2c00449

**Published:** 2022-12-19

**Authors:** Zerin Alimajstorovic, Susan P. Mollan, Olivia Grech, James L. Mitchell, Andreas Yiangou, Mark Thaller, Hannah Lyons, Matilde Sassani, Senali Seneviratne, Thomas Hancox, Andris Jankevics, Lukáš Najdekr, Warwick Dunn, Alexandra J. Sinclair

**Affiliations:** † Institute of Metabolism and Systems Research, College of Medical and Dental Sciences, University of Birmingham, Birmingham B15 2TT, U.K.; ‡ Birmingham Neuro-Ophthalmology, Queen Elizabeth Hospital, University Hospitals Birmingham, Birmingham B15 2WB, U.K.; § Department of Neurology, Queen Elizabeth Hospital, University Hospitals Birmingham NHS Foundation Trust, Birmingham B15 2WB, U.K.; ∥ School of Biosciences, University of Birmingham, Birmingham B15 2TT, U.K.; ⊥ Phenome Centre Birmingham, University of Birmingham, Birmingham B15 2TT, U.K.; # Institute of Molecular and Translational Medicine, Palacký University Olomouc, Hněvotínská 5, Olomouc 77900, Czech Republic; ¶ Department of Biochemistry and Systems Biology, Institute of Systems, Molecular, and Integrative Biology, University of Liverpool, Liverpool L69 7ZB, U.K.; ∇ Centre for Endocrinology, Diabetes and Metabolism, Birmingham Health Partners, Birmingham B15 2TT, U.K.

**Keywords:** idiopathic intracranial hypertension, intracranial pressure, metabolomics, arginine metabolism, lipid metabolism

## Abstract

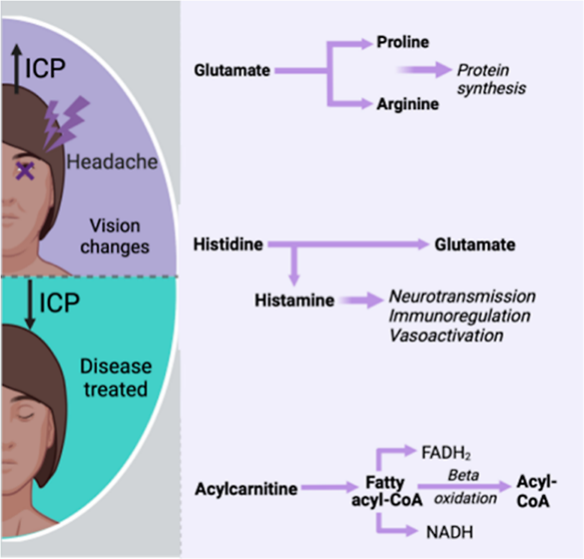

*Background*: Idiopathic intracranial hypertension (IIH) is characterized by increased intracranial pressure occurring predominantly in women with obesity. The pathogenesis is not understood. We have applied untargeted metabolomic analysis using ultrahigh-performance liquid chromatography–mass spectrometry to characterize the cerebrospinal fluid (CSF) and serum in IIH compared to control subjects. *Methods and findings*: Samples were collected from IIH patients (*n* = 66) with active disease at baseline and again at 12 months following therapeutic weight loss. Control samples were collected from gender- and weight-matched healthy controls (*n* = 20). We identified annotated metabolites in CSF, formylpyruvate and maleylpyruvate/fumarylpyruvate, which were present at lower concentrations in IIH compared to control subjects and returned to values observed in controls following weight loss. These metabolites showed the opposite trend in serum at baseline. Multiple amino acid metabolic pathways and lipid classes were perturbed in serum and CSF in IIH alone. Serum lipid metabolite pathways were significantly increased in IIH. *Conclusions*: We observed a number of differential metabolic pathways related to amino acid, lipid, and acylpyruvate metabolism, in IIH compared to controls. These pathways were associated with clinical measures and normalized with disease remission. Perturbation of these metabolic pathways provides initial understanding of disease dysregulation in IIH.

## 1 Introduction

Idiopathic intracranial hypertension (IIH) is a disease characterized by increased intracranial pressure (ICP), and the prevalence of IIH is higher in women with obesity,^1,2^ with a fourfold higher incidence in women than men.^3^ IIH incidence is increasing markedly (350% increase in the last decade), driven in part by the global obesity epidemic.^3,4^ A diagnosis of IIH is made where there is a combination of increased ICP without hydrocephalus (or space occupying the lesion in the brain) and normal cerebrospinal fluid (CSF) composition with no underlying cause identified.^5^ There is clear evidence that weight loss lowers ICP and induces remission of IIH.^6,7^ IIH causes blindness (in up to 24% of individuals) and long-term disabling headaches.^8,9^

The underlying etiology of IIH is unknown.^10^ Historically, IIH was considered a disease isolated to the central nervous system; however, evidence is emerging indicating that the disease also has a systemic phenotype.^11−14^ IIH patients are noted to have a number of features consistent with a metabolic syndrome including preferential truncal adiposity, doubled cardiovascular risk in comparison to women of similar age and body mass index (BMI), and insulin resistance with a greater magnitude of derangement to that mediated by obesity.^11,13^ Moreover, adipose tissue in IIH is transcriptionally and metabolically primed toward depot-specific lipogenesis,^11^ and steroid hormone analysis reveals a unique phenotype of serum and CSF androgen excess.^12^ However, the mechanisms driving the ICP dysregulation in IIH remain elusive and have been an ongoing area of research. Understanding the underlying cause and the development of disease specific biomarkers are key research priorities in IIH.^15^

Untargeted ultrahigh-performance liquid chromatography–mass spectrometry (UHPLC–MS) metabolomics has been successfully utilized to identify metabolic pathway derangements in a number of neurological disorders though evaluation of serum, plasma, and CSF.^16−19^ However, this approach, as well as the use of ^1^H NMR, has not been utilized in IIH to date. ^1^H NMR and mass spectrometry are different platforms that can achieve complementary analysis, which leverages the analytical advantages of each technology, but it is important to note that UHPLC–MS can detect a broader range of metabolites (10–30-fold more) and therefore provides a more comprehensive picture of metabolism.^20^

In this study, we aimed to initially investigate the systemic (as reported in serum) and central nervous system (as reported in CSF) metabolome of IIH female patients compared to BMI- and gender-matched control subjects and the relationship of differential metabolites with disease clinical features. Subsequently, we sought to evaluate longitudinal changes in the IIH metabolome driven by disease treatment. To achieve the research objectives, IIH patients and control subjects were assessed at baseline to identify IIH-specific metabolic phenotypes in serum and CSF. Subsequently, IIH patients were also evaluated at 12 months following weight loss therapy (surgical and dietary interventions) to assess longitudinal changes. Changes in the metabolic profiles of those achieving disease remission (ICP < 25 cmCSF responders) versus those who remained active (ICP ≥ 25 cmCSF responders) were also evaluated. Finally, we investigated the association of metabolomic profiles with clinical assessments to evaluate pathways related to disease clinical features. We noted a number of consistently differential metabolite pathways in IIH compared to controls, particularly those involving amino acid and lipid metabolism. These pathways were also associated with disease clinical features and altered over 12 months in line with disease remission. Perturbation of these metabolic pathways provides initial understanding of disease dysregulation in IIH.

## 2 Materials and Methods

### 2.1 Study Approval

A case-control study identified and recruited IIH subjects from neurology and ophthalmology clinics from five United Kingdom National Health Service hospitals. The clinical trial protocol and results have been published elsewhere,^7,21,22^ and control patients were recruited via advertisement on social media. Ethical approvals were from The National Research Ethics Committee West Midlands—The Black Country REC (14/WM/0011, Dudley, United Kingdom). Written informed consent was obtained from all participants in the study.

### 2.2 Study Population

Women aged 18–55 years, with a BMI ≥35 kg/m^2^ and active IIH [lumbar puncture opening pressure (LP OP) > 25 cmCSF and Frisén papilledema grade ≥1] on the date of research assessment visit, were recruited (detailed eligibility and exclusion criteria have been published^7,21^). The IIH participants attended for a trial visit at baseline and at 12 months, according to the published protocols.^21^ Following the baseline assessment, IIH patients were randomized 1:1 to either a WeightWatchers program (community weight management intervention) or a bariatric surgery pathway (gastric band, gastric sleeve, or Roux-en-Y gastric bypass^7,21^). Patients who had previously failed pharmacotherapy (such as acetazolamide) or failed community weight management were included in the study, provided they still met the eligibility criteria with ongoing active IIH. Control patients met the same inclusion and exclusion criteria applied to the IIH patients but were only evaluated at baseline.

### 2.3 Clinical Assessments

All participants underwent detailed medical history and clinical examination. BMI was calculated from weight and height using the following formula: BMI = (weight [kg]/height [m]^2^). Visual tests performed included the perimetric mean deviation (PMD) using Humphrey 24-2 Swedish Interactive Thresholding Algorithm (SITA) central threshold automated perimetry (Carl Zeiss Ltd, Cambridge, United Kingdom) and spectral domain optical coherence tomography (OCT; Spectralis, Heidelberg Engineering) to evaluate the average peripapillary retinal nerve fiber layer (RNFL). Ophthalmology measures (presence or absence of papilledema) were assessed by experienced clinicians (neuro-ophthalmologists) on the day of enrolment prior to any further test being conducted.^7,21^ Data from the most severely affected eye (as defined by PMD at baseline) were reported. Monthly headache days and headache severity were recorded using headache diaries, and headache-associated disability was measured using the headache impact test-6 score (HIT 6).

### 2.4 Sample Collection

**Figure 1:**
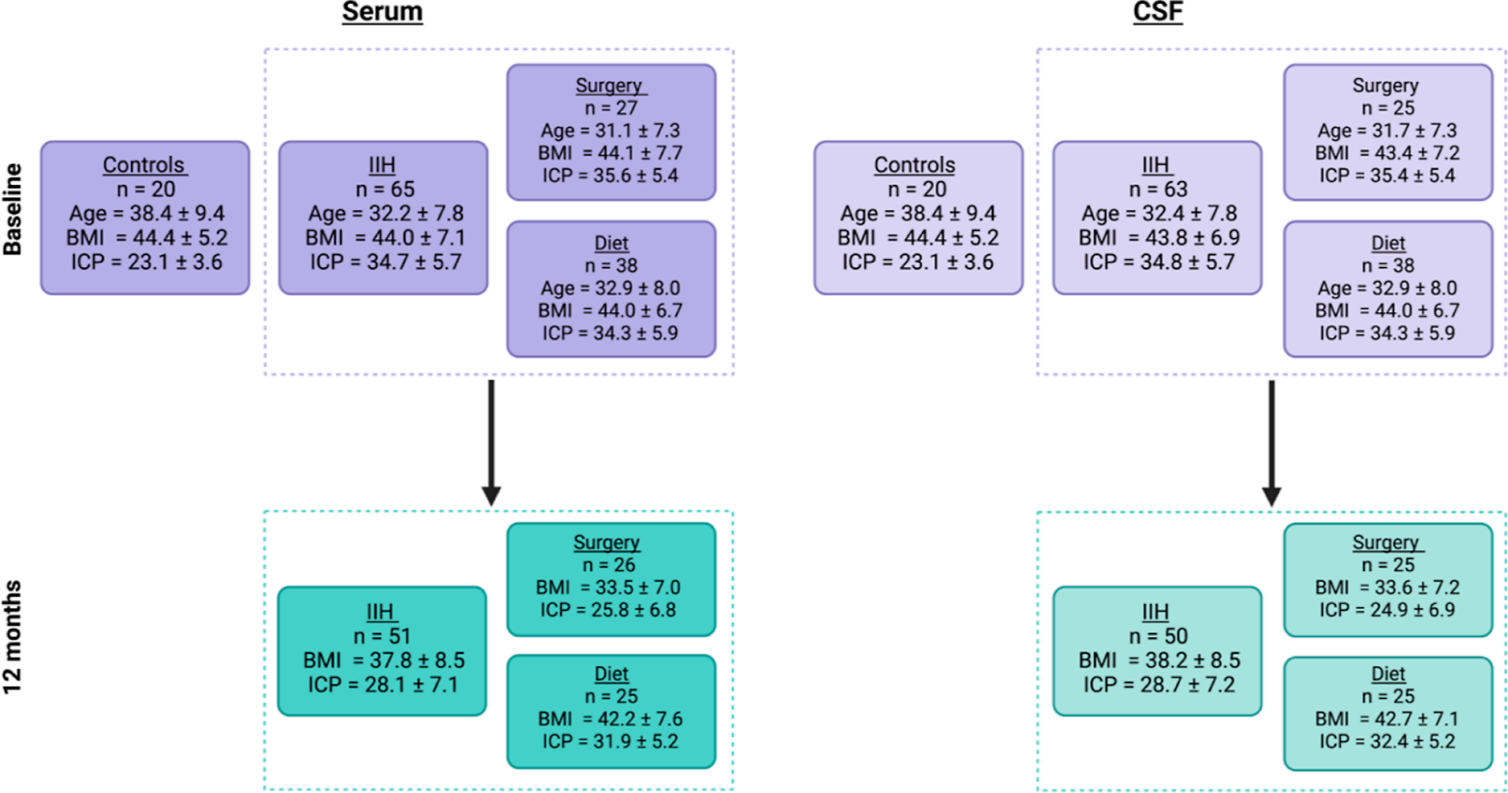
Schedule of assessment and consort diagram. IIH: idiopathic intracranial hypertension, BMI: body mass index, ICP: intracranial pressure, and CSF: cerebrospinal fluid.

All blood samples were collected following an overnight fast (from midnight). LP was conducted in the left lateral decubitus position under ultrasound guidance with knees bent at a 90° angle or more, and LP OP recorded before CSF was collected (up to 15 mL). Samples not analyzed immediately were centrifuged (10 min at 1500*g* at 4 °C), aliquoted, and stored at −80 °C. CSF samples were centrifuged (800*g* for 10 min at 4 °C), and the supernatant was aliquoted and stored at −80 °C. All samples processed only underwent a single freeze–thaw cycle before metabolomics analysis (Figure 1).

### 2.5 Metabolomics Analysis

Serum and CSF samples were prepared using a monophasic solvent extraction and analyzed by applying hydrophilic interaction liquid chromatography-based UHPLC–MS assays in positive- and negative-ion modes. Raw data were processed by applying XCMS^23^ to construct data matrices for each sample type and assay, and data were filtered and quality-assessed by applying QC sample data and subsequently analyzed by applying univariate statistical methods. Metabolite annotation was performed by applying MS1 and MS/MS data and matching them to publicly available databases (HMDB, LipidMaps, and KEGG) and mass spectral libraries (mzCloud). All methods applied are fully described in Supporting Information File 1.

### 2.6 Statistical Analysis, Correlation Analysis, and Pathway Enrichment Analysis—Serum and CSF

Statistical, correlation, and pathway enrichment analysis was performed in MetaboAnalyst v5.0.^24^ For statistical analysis, data were normalized to total sample response and log_10_-transformed. For correlation analysis, data were normalized to total sample response. Statistical analysis applied Student’s *t*-test for unpaired and paired analysis, and correlation analysis applied Spearman rank analysis. Fold changes were calculated using the mean of each class being studied. Pathway enrichment analysis applied pathway analysis, hypergeometric test (enrichment method), relative-betweenness centrality (topology analysis), and *Homo sapiens* (KEGG) as the pathway library. We applied the Benjamini–Hochberg procedure^25^ to correct for multiple testing.

We have reported statistical results after correction for multiple testing by applying the Benjamini–Hochberg procedure (because few thousands of tests have been performed) where metabolites were statistically significant after correction for multiple testing. In comparisons where no metabolites were statistically significant after correction for multiple testing, the statistical results were reported where no correction for multiple testing had been performed. Results which were corrected for multiple testing should be viewed as more statistically robust and of higher biological importance. Results which were not corrected for multiple testing should be viewed as less statistically robust but provide some potentially important biological conclusions, particularly were the results cluster in specific metabolic pathways and suggest further biological testing and validation and may be of interest to other scientists.

## 3 Results

### 3.1 Subject Characteristics

The characteristics of the subjects at baseline are described in Table 1. They were all women of which there were controls (*n* = 20) and those with active IIH (*n* = 60). Both groups were matched for BMI. Age was higher in the control cohort (mean ± SD age in controls = 38 ± 9.4 vs IIH = 32 ± 7.8, *p* = 0.003, Table 1).

**Table 1:** Characteristics of IIH and Control Subjects^a^

baseline characteristics	control	IIH
number (*n*)	20	66
age (years)*	38 ± 9.4	32 ± 7.8
BMI (kg/m^2^)	44.4 ± 5.2	44.0 ± 7.1
lumbar puncture opening pressure (cmCSF)*	23.1 ± 3.6	34.7 ± 5.7
perimetric mean deviation (worst eye) (dB)*	–2.8 ± 5.0	–4.1 ± 4.4
papilledema [OCT average retinal nerve fiber layer (worst eye)] (μm)*	96.6 ± 9.0	155.3 ± 97.9
monthly headache days*	10.8 ± 9.8	21.9 ± 8.4
headache severity per week*	2.3 ± 2.4	4.3 ± 2.5
headache disability HIT-6 score*	51.4 ± 10.4	64.7 ± 7.3

a Data presented as mean ± SD. * indicates a significant difference between groups as determined by the unpaired *t*-test (*p* < 0.05). BMI: body mass index, LP OP: lumbar puncture opening pressure, OCT: optical coherence tomography, and HIT-6: headache impact test-6.

### 3.2 CSF and Serum Metabolomes Differ between IIH Patients and Control Subjects

**Figure 2:**
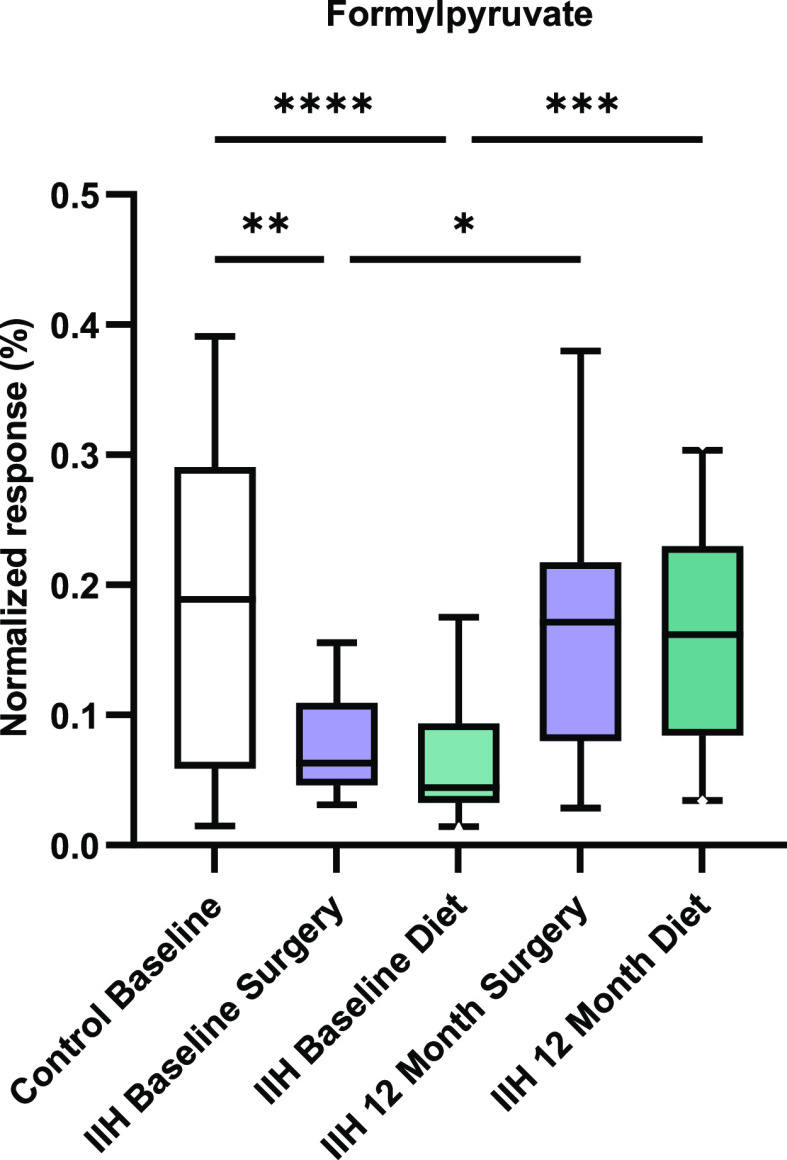
Relative concentration changes in formylpyruvate observed in CSF for control subjects (*n* = 18) and IIH subjects at baseline (surgery *n* = 15 and diet *n* = 29) and 12 months after surgical (*n* = 18) and dietary (*n* = 21) interventions.

**Figure 3:**
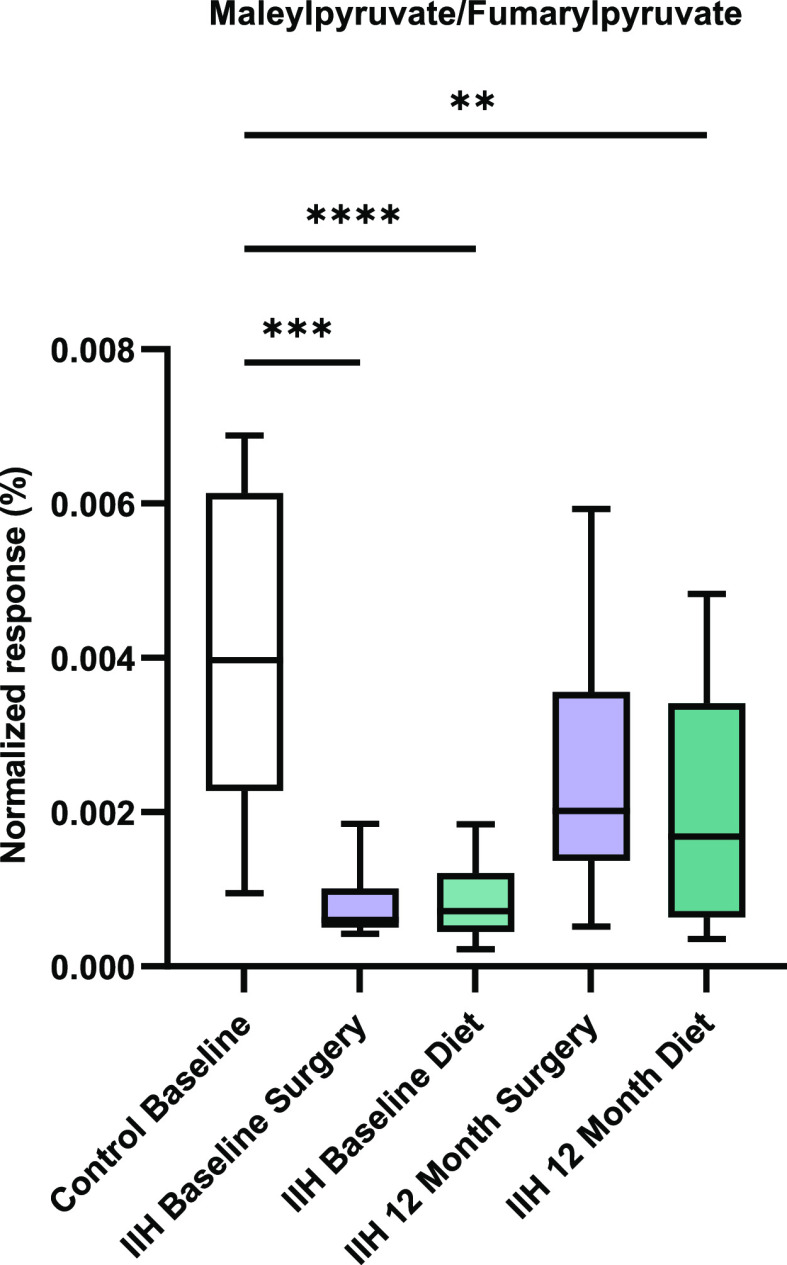
Relative concentration changes in isomers such as maleylpyruvate and fumarylpyruvate observed in CSF for the control (*n* = 13) and IIH at baseline (surgery *n* = 7 and diet *n* = 10) and 12 months after surgical (*n* = 14) and dietary (*n* = 18) interventions.

No individual metabolites were differential in both CSF and serum (*q* < 0.05, corrected for multiple testing). Comparison of CSF collected from IIH patients (*n* = 62) and matched controls (*n* = 18) at baseline highlighted two annotated metabolite features whose relative concentrations were lower in IIH compared to the control (*q* < 0.05, corrected for multiple testing; Supporting Information File 2). These two annotated metabolites were formylpyruvate (present at 2.7 times lower relative concentration in IIH subjects, Figure 2) and the isomers maleylpyruvate and/or fumarylpyruvate (present at 8.2 times lower relative concentration in IIH subjects, Figure 3). The same comparison applied to serum (IIH *n* = 65, controls *n* = 20) highlighted 21 annotated metabolite features whose concentration was differential (*q* < 0.05, corrected for multiple testing; Supporting Information File 3). In serum, formylpyruvate (2.5 times higher relative concentration in IIH subjects, *p* < 0.005, not corrected for multiple testing), three riboflavin (vitamin B_2_) metabolites, three panthethine-related metabolites, and a number of lipid classes that focused on fatty acid metabolism (acyl carnitines, diacylglycerides, fatty acids, glycerophospholipids and lysoglycerophospholipids) were also perturbed (*p* < 0.05, not corrected for multiple testing; Supporting Information File 4).

### 3.3 Metabolites Are Correlated with Clinical Parameters of IIH

#### 3.3.1 In the CSF Metabolome at Baseline

In the CSF of subjects with IIH, correlations between annotated metabolite features and clinical parameters (*p* < 0.05, not corrected for multiple testing) were observed for LP OP, PMD worst eye, papilledema as measured by OCT, headache frequency, headache severity, and HIT-6 headache disability (see Supporting Information Files 5–10). No metabolites were correlated in three or more of the six clinical parameters studied.

Subsequently, pathway enrichment analysis was performed for metabolites which correlated with each clinical parameter separately (*p* < 0.05, not corrected for multiple testing; Supporting Information File 11). Four enriched pathways were observed for the PMD worst eye, and these were vitamin B_6_ metabolism, arginine/proline metabolism, nitrogen metabolism, and glutamine/glutamate metabolism. No other clinical parameters showed statistically enriched metabolic pathways.

#### 3.3.2 In the Serum Metabolome at Baseline

For serum, correlations between metabolites and clinical parameters were observed for LP OP, PMD worst eye, papilledema as measured by OCT, headache frequency, headache severity, and HIT-6 headache disability (*p* < 0.05, not corrected for multiple testing; Supporting Information Files 12–17).

Glucose and hexacosahexaenoic acid were correlated with headache frequency, headache severity, and HIT-6 headache disability, but no other metabolites were correlated with three or more clinical parameters of IIH. Lipid classes which were perturbed in IIH patients when compared to control subjects were also observed to be associated with the clinical parameters and included acyl carnitines, diacylglycerides, glycerophospholipids, and lysoglycerophospholipids. Subsequently, pathway enrichment analysis was performed for metabolites which correlated for each clinical parameter separately (*p* < 0.05, not corrected for multiple testing; Supporting Information Files 18–23). The tricarboxylic acid cycle, the glyoxylate and dicarboxylate metabolism pathway, and histidine metabolism were enriched in two of the six parameters. All other reported metabolic pathways were enriched in only one clinical parameter class.

### 3.4 Metabolic Changes Related to Disease Changes Following a Weight-Loss Intervention

At 12 months following weight loss (surgery or diet), serum and CSF samples were collected from the IIH participants for comparison (*n* = 51 serum and *n* = 50 CSF). Those in the bariatric surgical program had significant therapeutic improvement in ICP (−10.1 ± 5.8 cmCSF) and clinical parameters compared to those on the community weight loss intervention (−2.1 ± 5.6 cmCSF). The clinical trial results are published elsewhere.^7^

#### 3.4.1 Metabolic Changes in the CSF Metabolome over 12 months

Paired statistical analysis of the CSF data at baseline and 12 months in those in the surgical cohort revealed that no annotated metabolite features were statistically significant (*q* < 0.05, corrected for multiple testing). In the diet group, three metabolite features were statistically significant (*q* < 0.05, corrected for multiple testing; Supporting Information File 24), including formylpyruvate and maleylpyruvate/fumarylpyruvate. Formylpyruvate and maleylpyruvate/fumarylpyruvate were statistically significant in the surgery group also but only before correction for multiple testing (*p* < 0.0002, not corrected for multiple testing; Supporting Information File 25).

#### 3.4.2 Metabolic Changes in the Serum Metabolome over 12 months

Paired analysis of the serum data at baseline and 12 months revealed 122 annotated metabolites which were statistically significant in the surgery group (*q* < 0.05, corrected for multiple testing; Supporting Information File 26).

Paired analysis of the serum data at baseline and 12 months in the diet group revealed no metabolites which were significant (*p* < 0.05, corrected for multiple testing). Overall, metabolites were only altered in the surgical cohort (those with the significant therapeutic reduction in ICP) and included acyl carnitines, fatty acids, glycerophospholipids, and lysoglycerophospholipids.

### 3.5 Changes in Metabolites Related to Disease Remission Compared to Those Not in Remission at 12 months

Metabolic changes were assessed between those achieving disease remission (ICP < 25 cmCSF at 12 months) and those who had ongoing active disease (ICP ≥ 25 cmCSF at 12 months). In CSF, no metabolites were statistically significant (*p* < 0.05, corrected for multiple testing). Formylpyruvate and maleylpyruvate/fumarylpyruvate were statistically significant when comparing remission to active disease in CSF with no correction for multiple testing (*p* < 0.05; Supporting Information File 27). In serum, 12 metabolites were statistically significant (*q* < 0.05, corrected for multiple testing; Supporting Information File 28). Neither formylpyruvate nor maleylpyruvate/fumarylpyruvate was present at different relative concentrations in the remission group compared to the active group in serum. Acyl carnitines, fatty acids, glycerophospholipids, and lysoglycerophospholipids were perturbed in serum in the remission versus non-remission groups (*p* < 0.05, not corrected for multiple testing; Supporting Information File 29).

### 3.6 Changes in ICP and Metabolite Relative Concentrations over 12 months Were Associated

We further investigated whether the change in ICP measurement from baseline to 12 months was correlated to the change in relative concentration of metabolites from baseline to 12 months in the entire IIH cohort. In CSF and serum, no metabolites were correlated with ICP change after results were corrected for multiple testing (*q* < 0.05). In the serum analysis, multiple fatty acids, glycerophospholipids, and lysoglycerophospholipids were observed to be correlated with the change in relative concentration in ICP between baseline and 12 months with no correction applied for multiple testing (*p* < 0.05; Supporting Information File 30). Neither formylpyruvate nor maleylpyruvate/fumarylpyruvate was associated with changes in ICP between baseline and 12 months in CSF or serum (*p* < 0.05, not corrected for multiple testing).

## 4 Discussion

Utilizing untargeted UHPLC–MS, we have characterized the metabolite profiles of IIH subjects in serum and CSF, in comparison to BMI- and gender-matched controls. Importantly, we have also evaluated changes in metabolite profiles occurring with disease treatment through 12 months of weight loss intervention and further evaluated the metabolite changes with disease remission. We were able to investigate the association of water-soluble metabolites and lipids with a diagnosis of IIH, the associated clinical measurements, and longitudinal disease course following bariatric surgical intervention (which significantly reduced ICP and treated IIH) and a dietary intervention (which did not significantly reduce ICP and treat IIH). Metabolic pathways and lipids have been observed to be repeatedly perturbed in separate comparisons of (1) disease versus control, (2) in association with IIH clinical measurements, and (3) in association with disease activity. Amino acid metabolism (including arginine and proline metabolism and histidine metabolism) and lipid classes including acyl carnitines, fatty acids, glycerophospholipids, and lysoglycerophospholipids have been observed to be perturbed. No previous studies have identified these metabolic pathways and lipid classes, and we suggest that they may play a pathological role in IIH. The identified areas of metabolism would be valuable to interrogate in future mechanistic studies in probing disease etiology and exploring the role of obesity in the development of IIH.

Formylpyruvate and the isomers maleylpyruvate and/or fumarylpyruvate were observed to be present at different concentrations in serum and CSF when comparing IIH to control subjects. The maleylpyruvate/fumarylpyruvate feature could on first appearance be a sodium adduct of a formylpyruvate dimer based on *m*/*z* measurements. However, the author has discounted this because the two features are not correlated (*r* = 0.10, *p* > 0.05; Supporting Information File 31); in metabolite annotation, we assume that two features from the same metabolite are being reported when *r* > 0.50, and the chromatographic peak shapes are not the same as would be expected for different features of the same metabolite. At baseline in CSF, formylpyruvate and maleylpyruvate/fumarylpyruvate were present at lower concentrations in IIH compared to control subjects. These concentrations increased to levels similar to control subjects following bariatric surgery (but not after a diet intervention). Although these metabolites were not correlated with clinical parameters, the alterations documented were potentially related to disease activity as concentrations normalized with effective treatment and disease remission. In serum, formylpyruvate was present at higher concentrations in IIH baseline compared to control subjects, and in these patients, the metabolite decreased in concentration following bariatric surgery. Of significant interest was the finding that the altered metabolic pathways were distinct in serum and CSF, with changes in lipid classes in serum mostly not observed in CSF and vice versa. This suggests that different pathways are up- and down-regulated simultaneously systemically and in the central nervous system.

Both maleylpyruvate and fumarylpyruvate are metabolically linked as acylpyruvates. Formylpyruvate is metabolized by gut microbes,^26,27^ whereas maleylpyruvate and fumarylpyruvate are involved in tyrosine metabolism in humans^28^ (although could also be metabolized by gut microbes). Recently, acylpyruvates have been recognized to be metabolized by fumarylacetoacetate hydrolase domain-containing protein 1 which exhibits acylpyruvate hydrolase activity and is localized in mitochondria.^29^ The observed relative concentration of formylpyruvate was increased in serum and decreased in CSF when comparing IIH patients to controls, suggesting that this metabolite is synthesized outside the central nervous system (e.g., human systemic tissues or gut microbiome) and then potentially crosses the blood–brain barrier. The increase in formylpyruvate in serum could be related to alterations in the gut microbiome, which have recently be shown to be important in IIH development.^30^ Notably, other metabolite pathways that were found to be affected in this study have also been shown to be altered by gut microbiota including acyl carnitines, diacylglycerides, and sphingolipids.^31−34^ One explanation is that in the disease state, with increased ICP, the transfer of formylpyruvate across the blood–brain barrier appears to be reduced and following therapeutic interventions, and reduction of ICP, transport across the blood–brain barrier recovers and formylpyruvate returns to nearer non-disease levels in CSF and serum. The same process may be in operation for maleylpyruvate/fumarylpyruvate as the same trends are observed and the transporters involved are the same. Since this finding persists following multi-comparison testing and changes relate strongly with response to treatment, it is unlikely that this finding is a statistical artifact.

### 4.1 Lipid Metabolism

Obesity and recent weight gain are known major risk factors for IIH, and significant weight loss through surgical or dietary interventions has been shown to be a disease-modifying treatment of IIH.^5−7^ Changes in the relative concentrations of lipids including acyl carnitines, diacylglycerides, fatty acids, glycerophospholipids, and lysoglycerophospholipids have previously been associated with obesity compared to non-obese populations.^35−38^ Changes in acyl carnitines, fatty acids, and oxidized fatty acids are associated with obesity including changes linked to mitochondrial dysfunction and increased oxidative stress in the mitochondria.^39−48^ The BMIs of the IIH patients and control subjects were matched, so observed changes suggest differences in lipid metabolism in IIH patients independent of obesity. Changes in lipid species in these classes were also observed in response to surgical interventions and differentiated disease remission subjects from those with ongoing active disease in serum (but not CSF). This is an important finding and may have practical clinical implications in understanding the role of weight loss in modifying ICP in IIH. We observed that changes in these lipid classes (when comparing IIH patients to control subjects) were associated with the following clinical parameters of IIH: PMD (a marker of visual function), OCT RNFL thickness (a measure of papilledema), HIT-6 score (headache disability), and headache severity. These changes suggest a systemic change in fatty acid metabolism potentially through fatty acid β-oxidation in the mitochondria.^49,50^ Perturbed mitochondrial function has been associated with headache generation and may contribute to IIH.^8,51−53^

### 4.2 Amino Acid Metabolism

Pathway enrichment analysis identified a number of amino acid metabolic pathways in serum which were associated with clinical symptoms. These included alanine/aspartate/glutamate metabolism and histidine metabolism. Histidine metabolism has also been associated with headache, since a catabolic product of histidine, histamine, plays a mechanistic role in migraine development and anti-histamine medications have migraine therapeutic properties.^54−56^ Histamine is involved in immune response and is a neurotransmitter linked to vasodilation and reduction in blood pressure.^57,58^

In CSF, pathway enrichment analysis identified arginine/proline metabolism and glutamate/glutamine metabolism as associated to one clinical symptom (MD worst eye). Arginine metabolism has previously been linked to the development and treatment of migraines through its role in the synthesis of nitric oxide.^59−70^ Nitric oxide is synthesized from arginine by the endothelial nitric oxide synthase and synthesizes citrulline as a byproduct.^71,72^ Nitric oxide is important in smooth muscle relaxation, vasodilation, and increased blood flow and is a potent headache provocation agent.^72^ It is possible that the perturbed arginine metabolism with downstream effects on nitric oxide may be linked to the severe headache phenotype in IIH.^73^ The arginine metabolism derangement may also reflect obesity in IIH as nitric oxide is associated with metabolic changes in obesity and diabetes including increased inflammation and oxidative stress in obese women.^59,71,74,75^

**Figure 4:**
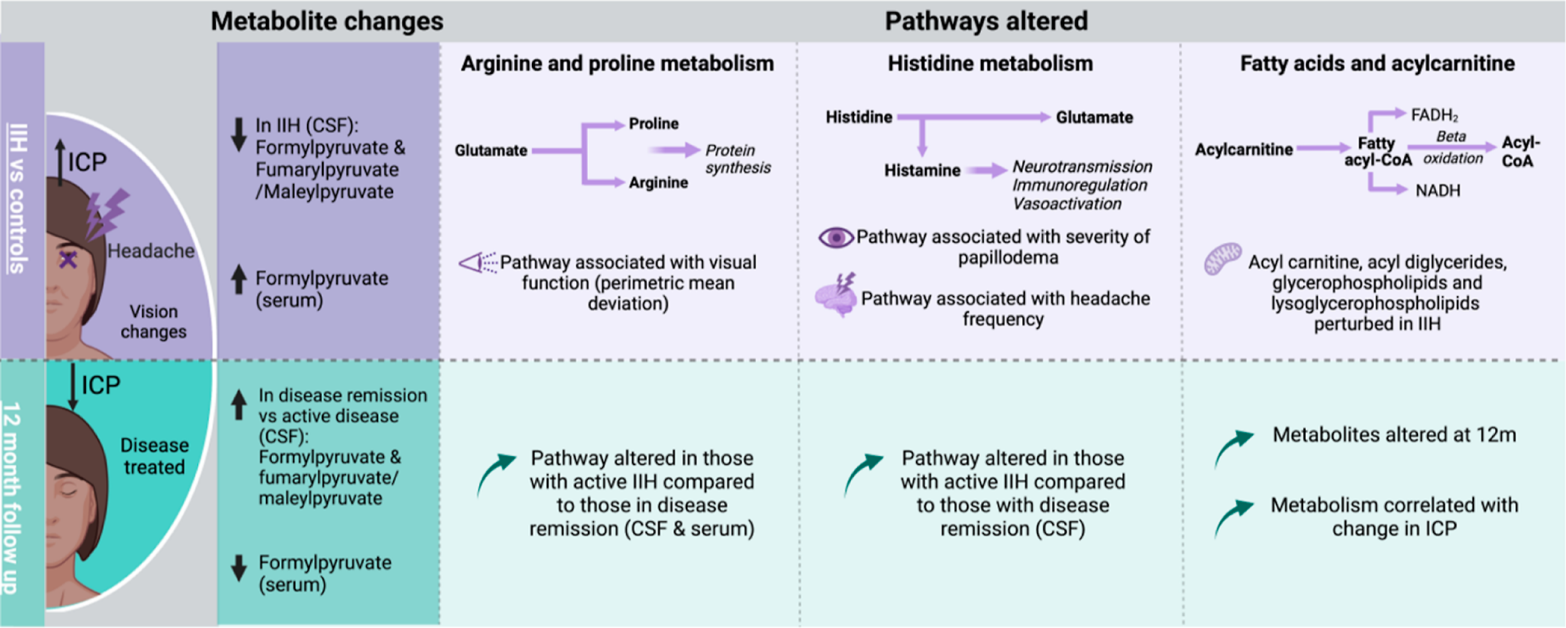
Infographic underlying the metabolite changes and pathways altered between IIH patients vs control subjects and at 12 month follow up. IIH: idiopathic intracranial hypertension, ICP: intracranial pressure, and CSF: cerebrospinal fluid.

The study performed does not allow us to define whether the metabolic changes reported are a cause of IIH or a consequence of IIH, and further studies are required to dissect the cause and effect and pathophysiological mechanisms (Figure 4).

### 4.3 Study Limitations

There are some limitations to the reported study which should be considered. Due to the rarity of the disease, there were relatively small numbers of participants in the IIH cohort; however, this sample size is large compared to other IIH studies. The control group was also small due to the challenge of recruiting healthy obese volunteers for a lumbar puncture, and this may have reduced our ability to robustly identify small but important metabolic changes. Additionally, we prioritized matching for BMI as this was determined as the major confounder. However, age was not matched (mean IIH age 32 vs mean control age 38 years), and this may have impacted the results. Of note, no metabolites discussed were found to be correlated with age. It should also be noted that our post-surgical samples were collected at the 12 month visit during the study, and bariatric surgery took place at an individual time point between 3 and 12 months (mean 4 months). This could impact the results and also means that we were unable to examine the sustainability of changes and how these could potentially impact on the longer-term effects of each intervention. Re-evaluation at 2 and 5 years post-surgery would be of interest. We note that previous studies with serial sampling have demonstrated that some of the changes seen after bariatric surgery are transient.^76^

Some metabolites are identified based on comparison of retention time and/or MS/MS data to data collected for authentic chemical standards, although other metabolites are annotated without comparison to chemical standards. For this reason, we have applied pathway enrichment analysis to reduce (but not fully eliminate) the probability of false positive conclusions. For example, if eight metabolites are statistically significant and present in a single pathway, then we have more confidence that this is a biologically valid conclusion compared to deriving biological conclusions from a single statistically significant metabolite without applying pathway enrichment analysis.

## 5 Conclusions and Future Directions

The etiology of IIH is poorly understood. We noted multiple differential metabolite pathways in IIH compared to controls, predominantly those involving amino acid and lipid metabolism. These pathways were also associated with disease clinical features and altered over 12 months in line with disease remission. Previous studies of metabolism in IIH have focused on steroid hormone metabolism and metabolite quantification using NMR. Therefore, this discovery-based approach was chosen to investigate global metabolism with the goal of identifying metabolic targets requiring further study. The perturbed pathways identified here provide initial insights into disease metabolic flux and are a focus for future mechanistic evaluation in IIH.

## Supporting Information Available

Table of contents (PDF)
Additional metabolomics experimental details, materials, and methods including chemicals and solvents, sample preparation—serum and CSF, UHPLC–MS analysis—serum, UHPLC–MS analysis—CSF, raw data processing and quality assessment—serum and CSF, metabolite annotation—serum and CSF, and references (PDF)
UHPLC–MS raw data for all serum and CSF metabolites including comparison of CSF collected from IIH patients and matched controls at baseline, comparison of serum collected from IIH patients and matched controls at baseline, comparison of serum collected from IIH patients and matched controls at baseline (not corrected for multiple testing), correlation between annotated metabolite features and clinical parameter LP OP at baseline, correlation between annotated metabolite features and the clinical parameter PMD worst eye at baseline, correlation between annotated metabolite features and clinical parameter papilledema as measured by OCT at baseline, correlation between annotated metabolite features and clinical parameter headache frequency at baseline, correlation between annotated metabolite features and clinical parameter headache severity at baseline, correlation between annotated metabolite features and clinical parameter HIT-6 headache disability at baseline, pathway enrichment analysis for CSF metabolites which correlated with the clinical parameter PMD worst eye at baseline, correlation between annotated metabolite features and clinical parameter LP OP at baseline, correlation between annotated metabolite features and clinical parameter PMD worst eye at baseline, correlation between annotated metabolite features and clinical parameter papilledema as measured by OCT at baseline, correlation between annotated metabolite features and clinical parameter headache frequency at baseline, correlation between annotated metabolite features and clinical parameter headache severity at baseline, correlation between annotated metabolite features and clinical parameter HIT-6 headache disability at baseline, pathway enrichment analysis for serum metabolites which correlated with clinical parameter LP OP at baseline, pathway enrichment analysis for serum metabolites which correlated with the clinical parameter PMD worst eye at baseline, pathway enrichment analysis for serum metabolites which correlated with clinical parameter papilledema as measured by OCT at baseline, pathway enrichment analysis for serum metabolites which correlated with clinical parameter headache frequency at baseline, pathway enrichment analysis for serum metabolites which correlated with clinical parameter headache severity at baseline, pathway enrichment analysis for serum metabolites which correlated with clinical parameter HIT-6 headache disability at baseline, metabolic changes in the CSF metabolome of the diet cohort over 12 months, metabolic changes in the CSF metabolome of the surgery cohort over 12 months, metabolic changes in the serum metabolome of the surgery cohort over 12 months, changes in CSF metabolites related to disease remission compared to those not in remission at 12 months, changes in serum metabolites related to disease remission compared to those not in remission at 12 months, perturbed metabolites in serum of the remission versus non-remission groups, and metabolites correlated with the change in relative concentration in ICP between baseline and 12 months (XLSX)
Scatter plot visualizing the normalized peak areas (%) for two metabolite features (M115T44 and M253T44) to assess whether the two metabolite features are generated from the same or different metabolites (PDF)

## Notes

Role of Funder/Sponsor: The funders had no role in the design or conduct of the study; no role in collection, management, analysis, or interpretation of the data; no role in preparation, review, or approval of the manuscript; and no role in the decision to submit the manuscript for publication and in the design, execution, or write-up of this trial.

## Notes

Trial details: IIH:WT was funded by The National Institute for Health Research (NIHR-CS-011-028) and registered with ClinicalTrials.gov: NCT02124486.
