# Supplementary material for: Dysregulation of Amino Acid, Lipid, and Acylpyruvate Metabolism in Idiopathic Intracranial Hypertension: A Non-targeted Case Control and Longitudinal Metabolomic Study
Source: J Proteome Res. 2022 Dec 19;22(4):1127–37. doi: 10.1021/acs.jproteome.2c00449 (PMC10088035; doi:10.1021/acs.jproteome.2c00449)
Supplement: Supplementary file 1 — pr2c00449_si_001.pdf [file pr2c00449_si_001.pdf]

Supporting information cover sheet

Dysregulation of amino acid, lipid and acylpyruvate metabolism in idiopathic intracranial hypertension: A non-targeted case control and longitudinal metabolomic study

Zerin Alimajstorovic[1], Susan P Mollan[2], Olivia Grech[1], James L Mitchell[1,3], Andreas Yiangou[1,3], Mark Thaller[1,3], Hannah Lyons[1,3], Matilde Sassani[1,3], Senali Seneviratne[1], Thomas Hancox[4], Andris Jankevics[4,5], Lukáš Najdekr[4,5,6], Warwick Dunn[1,4,5,7] and Alexandra J Sinclair[1,3,8*]

Author Affiliations

1 Institute of Metabolism and Systems Research, College of Medical and Dental Sciences, University of Birmingham, Birmingham, B15 2TT, United Kingdom.

2 Birmingham Neuro-Ophthalmology, University Hospitals Birmingham, Queen Elizabeth Hospital, Birmingham, B15 2WB, UK

3 Department of Neurology, University Hospitals Birmingham NHS Foundation Trust, Queen Elizabeth Hospital, Birmingham, B15 2WB, UK.

4 School of Biosciences, University of Birmingham, Birmingham, B15 2TT, UK.

5 Phenome Centre Birmingham, University of Birmingham, Birmingham, B15 2TT, UK.

6 Institute of Molecular and Translational Medicine, Palacký University Olomouc, Hněvotínská 5, 77900 Olomouc, Czech Republic.

7 Department of Biochemistry and Systems Biology, Institute of Systems, Molecular, and Integrative Biology, University of Liverpool, Liverpool L69 7ZB, UK

8 Centre for Endocrinology, Diabetes and Metabolism, Birmingham Health Partners, Birmingham, B15 2TT, UK.

Supporting information table of contents

a) **Supplementary file 1:** Additional metabolomics experimental details, materials, and methods (PDF) including:

1. Chemicals and solvents
2. Sample preparation – serum and CSF
3. UHPLC-MS analysis – serum
4. UHPLC-MS analysis – CSF
5. Raw data processing and quality assessment - serum and CSF
6. Metabolite annotation - serum and CSF
7. References

b) **Supplementary data CSF and Serum file:** UHPLC-MS raw data for all serum and CSF metabolites listed in sheet order from supplementary file 2 (SF2) to supplementary file 30 (SF30) (Excel DOC), including:

1. SF2_CSF - Comparison of CSF collected from IIH patients and matched controls at baseline
2. SF3_Serum - Comparison of serum collected from IIH patients and matched controls at baseline
3. SF4_Serum - Comparison of serum collected from IIH patients and matched controls at baseline (not corrected for multiple testing)
4. SF5_CSF - Correlation between annotated metabolite features and clinical parameter LP OP at baseline
5. SF6_CSF - Correlation between annotated metabolite features and clinical parameter PMD worst eye at baseline
6. SF7_CSF - Correlation between annotated metabolite features and clinical parameter papilloedema as measured by OCT at baseline

7. SF8_CSF - Correlation between annotated metabolite features and clinical parameter headache frequency at baseline
8. SF9_CSF - Correlation between annotated metabolite features and clinical parameter headache severity at baseline
9. SF10_CSF - Correlation between annotated metabolite features and clinical parameter HIT-6 headache disability at baseline
10. SF_11 CSF - Pathway enrichment analysis for CSF metabolites which correlated with clinical parameter PMD worst eye at baseline
11. SF_12 Serum - Correlation between annotated metabolite features and clinical parameter LP OP at baseline
12. SF_13 Serum - Correlation between annotated metabolite features and clinical parameter PMD worst eye at baseline
13. SF_14 Serum - Correlation between annotated metabolite features and clinical parameter papilloedema as measured by OCT at baseline
14. SF_15 Serum - Correlation between annotated metabolite features and clinical parameter headache frequency at baseline
15. SF_16 Serum - Correlation between annotated metabolite features and clinical parameter headache severity at baseline
16. SF_17 Serum - Correlation between annotated metabolite features and clinical parameter HIT-6 headache disability at baseline
17. SF_18 Serum - Pathway enrichment analysis for serum metabolites which correlated with clinical parameter LP OP at baseline
18. SF_19 Serum - Pathway enrichment analysis for serum metabolites which correlated with clinical parameter PMD worst eye at baseline
19. SF_20 Serum - Pathway enrichment analysis for serum metabolites which correlated with clinical parameter papilloedema as measured by OCT at baseline

20. SF_21 Serum - Pathway enrichment analysis for serum metabolites which correlated with clinical parameter headache frequency at baseline
21. SF_22 Serum - Pathway enrichment analysis for serum metabolites which correlated with clinical parameter headache severity at baseline
22. SF_23 Serum - Pathway enrichment analysis for serum metabolites which correlated with clinical parameter HIT-6 headache disability at baseline
23. SF_24 CSF - Metabolic changes in the CSF metabolome of the diet cohort over 12 months
24. SF_25 CSF - Metabolic changes in the CSF metabolome of the surgery cohort over 12 months
25. SF_26 Serum - Metabolic changes in the serum metabolome of the surgery cohort over 12 months
26. SF_27 CSF - Changes in CSF metabolites related to disease remission compared to those not in remission at 12 months
27. SF_28 Serum - Changes in serum metabolites related to disease remission compared to those not in remission at 12 months
28. SF_29 Serum - Perturbed metabolites in serum of the remission vs non-remission groups
29. SF_30 Serum – Metabolites correlated with the change in relative concentration in ICP between baseline and 12 months

c) **Supplementary file 31:** Scatter plot visualising the normalised peaks areas (%) for two metabolite features (M115T44 and M253T44) to assess whether the two metabolite features are generated from the same or different metabolites.
