# Supplementary material for: Dysregulation of Amino Acid, Lipid, and Acylpyruvate Metabolism in Idiopathic Intracranial Hypertension: A Non-targeted Case Control and Longitudinal Metabolomic Study
Source: J Proteome Res. 2022 Dec 19;22(4):1127–37. doi: 10.1021/acs.jproteome.2c00449 (PMC10088035; doi:10.1021/acs.jproteome.2c00449)
Supplement: Supplementary file 2 — pr2c00449_si_002.pdf [file pr2c00449_si_002.pdf]

Supplementary File: Metabolomics methods

1. Chemicals and solvents

Acetonitrile, methanol and water (HPLC grade acetonitrile and water) were purchased from Fisher Scientific (Loughborough, U.K.). Formic acid (≥98.0% purity) was purchased from VWR International (Lutterworth, U.K.), and ammonium formate (≥98.0% purity) was purchased from Sigma-Aldrich (Poole, U.K).

2. Sample preparation – serum and CSF

Samples were extracted to separate low molecular weight metabolites from other biochemicals including proteins, RNA and DNA. Samples were thawed and extracted on ice. For serum, 150µL of acetonitrile/methanol – 3:1 (v/v) (LC–MS grade, LiChrosolv, Merck) was added to 50 µL of plasma, for CSF 400µL of acetonitrile/methanol – 1:1 (v/v) (LC–MS grade, LiChrosolv, Merck) was added to 100 µL of CSF. Samples were then vortex mixed (15s) followed by centrifugation (21 000*g*, 20 min, 4 °C) and transfer of 150µL (for serum) and 90µL (for CSF) aliquots in to a glass LC autosampler vial (VI-04-12-02RVG 300µL plastic, Chromatography Direct, UK). A single pooled QC sample was prepared by combining aliquots (10 µL serum, 27 µL CSF) of all biological samples and vortex mixing (2 min serum, 5 min CSF). Aliquots (50 µL serum, 100µL CSF) of the pooled QC sample were extracted as defined above. Two extraction blank samples were prepared by performing the extraction process in the absence of a biological sample or solvent.

3. UHPLC-MS analysis - serum

The samples were analysed applying a Dionex UltiMate 3000 Rapid Separation LC system (Thermo Fisher Scientific, MA, USA) coupled with a heated electrospray Q Exactive Focus mass spectrometer (Thermo Fisher Scientific, MA, USA). Sample extracts were analysed on a Accucore-150-Amide-HILIC column (100

× 2.1 mm, 2.6 µm, Thermo Fisher Scientific, MA, USA). Mobile phase A consisted of 10 mM ammonium formate and 0.1% formic acid in 95% acetonitrile/water and mobile phase B consisted of 10 mM ammonium formate and 0.1% formic acid in 50% acetonitrile/water. Flow rate was set to 0.50 $ml.min^{-1}$ with the following gradient: t = 0.0, 1% B; t = 1.0, 1% B; t = 3.0, 15% B; t = 6.0, 50% B; t = 9.0, 95% B; t = 10.0, 95% B; t = 10.5, 1% B; t = 14.0, 1% B, all changes were linear with curve = 5. The column temperature was set to 35 °C and the injection volume was 2 µL. Data were acquired in positive and negative ionisation modes separately within the mass range of 70–1050 *m/z* at resolution 70,000 (FWHM at *m/z* 200). Ion source parameters were set as follows: Sheath gas = 53 arbitrary units, Aux gas = 14 arbitrary units, sweep gas = 3 arbitrary units, Spray Voltage = 3.5 kV, Capillary temp. = 269 °C, Aux gas heater temp. = 438 °C. Data dependent MS/MS in 'Discovery mode' was used for the MS/MS spectra acquisition using following settings: resolution = 17,500 (FWHM at *m/z* 200); Isolation width = 3.0 *m/z*; stepped normalised collision energies (stepped NCE) = 25, 60, 100%. Spectra were acquired in three different mass ranges: 70–200 m/z; 200–400 m/z; 400–1000 m/z. QC samples were analysed as injections 1-11 with the exception of injection 5 which was an extraction blank sample, after every 6th biological sample injection and two QC samples were analysed at the end of the run followed by analysis of the extraction blank sample. QC injections 6-8 were applied for MS/MS data collection.

4. UHPLC-MS analysis - CSF

Samples were analysed separately in positive and negative ion modes using a Vanquish UHPLC coupled to an electrospray Q Exactive Plus mass spectrometer (Thermo Scientific, San Jose, CA, U.S.A.). The assay applied a hydrophilic interaction chromatography (HILIC) method which used a Thermo Accucore 150 Amide column (2.1 ×100 mm, 2.6 µm) with a flow rate of 500 $\mu L.min^{-1}$ and with two mobile phases, mobile phase A (10 mM ammonium formate in 95% acetonitrile/water (v/v) + 0.1% formic acid) and mobile phase B (10 mM ammonium formate in 50% acetonitrile/water (v/v) + 0.1% formic acid). A 14

min gradient elution was applied as follows: 0 min, 1% B; 1 min, 1% B; 3 min, 15% B; 6 min, 50% B; 9 min, 95% B; 10min, 95% B; 10.5 min, 1% B. A 2 µL injection volume was applied and a *m/z* range of 150−2000 Da was applied. Data were acquired in positive and negative ionisation modes separately within the mass range of 70–1050 *m/z* at resolution 70,000 (FWHM at *m/z* 200). Ion source parameters were set as follows: Sheath gas = 53 arbitrary units, Aux gas = 14 arbitrary units, sweep gas = 3 arbitrary units, Spray Voltage = 3.5 kV, Capillary temp. = 269 °C, Aux gas heater temp. = 438 °C. Data dependent MS/MS in 'Discovery mode' was used for the MS/MS spectra acquisition using following settings: resolution = 17,500 (FWHM at *m/z* 200); Isolation width = 3.0 *m/z*; stepped normalised collision energies (stepped NCE) = 25, 60, 100%. Spectra were acquired in three different mass ranges: 70–200 *m/z*; 200–400 *m/z*; 400–1000 *m/z*. QC samples were analysed as injections 1-11 with the exception of injection 5 which was an extraction blank sample, after every 6th biological sample injection and two QC samples were analysed at the end of the run followed by analysis of the extraction blank sample. QC injections 6-8 were applied for MS/MS data collection.

5. Raw data processing and quality assessment - serum and CSF

Vendor format raw data files (.RAW) were converted to the mzML file format using ProteoWizard software (1). Deconvolution was performed by XCMS software 4 (version 1.46 running in the Galaxy environment) (2). R package IPO (3) was used to guide, optimise and obtain XCMS peak picking parameters. Serum: XCMS was operated applying min peak width (4s); max peak width (30s); ppm (12); mzdiff (0.001); bw (0.25); mzwid (0.01); minfrac (0.5). CSF: XCMS was operated applying min peak width (4s); max peak width (30s); ppm (8); mzdiff (0.003); bw (4.25); mzwid (0.0157); minfrac (0.5). Obiwarp retention time correction was applied to CSF HILIC negative ion mode data only. A data matrix of peak areas for metabolite features (*m/z*-retention time pairs) vs. samples were constructed. Each data matrix was filtered as follows: re-equilibration QC samples were removed; any feature whose

median intensity in the biological samples was <20× its median intensity of the process blank samples was removed; any feature present in < 90% of the QC samples was removed; features with RSD ≥ 30% across the intra-study QC samples were removed; samples with >50% missing values were removed; features with >50% missing values were removed. The dataset showed UHPLC–MS variation in QC abundance across the run; hence, data matrices were corrected for run order drift in intensity using the Quality Control-Robust Spline Correction (QC-RSC) algorithm. These steps were executed using the R/Bioconductor package structToolbox (4).

6. Metabolite annotation - serum and CSF

Putative metabolite annotation applying MS1 data was performed by applying the Python package BEAMSpy (https://github.com/computational-metabolomics/beamspy); *m/z* values of all experimentally observed peaks were searched directly, one at a time, against the HMDB (https://hmdb.ca) (5) and LIPID MAPS (https://www.lipidmaps.org) (6) databases, and all matches within a 5 ppm mass error tolerance were recorded as according to the Metabolomics Standards Initiative (MSI) reporting standards – MSI level 3 were *m/z* only has been applied (7). Multiple annotations (e.g. isomeric compounds) could be observed for a single detected metabolite feature. Here, the term putatively annotated metabolite refers to either single or multiple chemical compounds with the same retention time and accurate *m/z*. To generate more robust compound annotations (MSI level 2), QC sample UHPLC−MS/MS data were matched to MS/MS databases using either LipidSearch software (lipid annotation based on in silico-predicted MS/MS mass spectra; version 4.2.18, Thermo Fisher Scientific) or Thermo Fisher Scientific Compound Discoverer 3 software (polar compound annotations using the mzCloud database: hits with an mzCloud best match score of >60% were retained; https://www.mzcloud.org). Compounds were also matched using their retention time to that of an authentic chemical standard analysed using the same chromatography conditions (retention time tolerance ± 5 s).

References

1. Kessner D, Chambers M, Burke R, Agus D, Mallick P. ProteoWizard: open source software for rapid proteomics tools development. Bioinformatics. 2008;24(21):2534-6.
2. Smith CA, Want EJ, O'Maille G, Abagyan R, Siuzdak G. XCMS: processing mass spectrometry data for metabolite profiling using nonlinear peak alignment, matching, and identification. Anal Chem. 2006;78(3):779-87.
3. Libiseller G, Dvorzak M, Kleb U, Gander E, Eisenberg T, Madeo F, et al. IPO: a tool for automated optimization of XCMS parameters. BMC Bioinformatics. 2015;16:118.
4. Lloyd GR, Jankevics A, Weber RJM. Struct: an R/bioconductor-based framework for standardised metabolomics data analysis and beyond. Bioinformatics. 2020;36(22-23):5551-2.
5. Wishart DS, Guo A, Oler E, Wang F, Anjum A, Peters H, et al. HMDB 5.0: the Human Metabolome Database for 2022. Nucleic Acids Res. 2022;50(D1):D622-D31.
6. Gateway TLML. 2022 [February 28, 2022]. Available from: https://www.lipidmaps.org/.
7. Sumner LW, Amberg A, Barrett D, Beale MH, Beger R, Daykin CA, et al. Proposed minimum reporting standards for chemical analysis Chemical Analysis Working Group (CAWG) Metabolomics Standards Initiative (MSI). Metabolomics. 2007;3(3):211-21.
