# Supplementary material for: Dysregulation of Amino Acid, Lipid, and Acylpyruvate Metabolism in Idiopathic Intracranial Hypertension: A Non-targeted Case Control and Longitudinal Metabolomic Study
Source: J Proteome Res. 2022 Dec 19;22(4):1127–37. doi: 10.1021/acs.jproteome.2c00449 (PMC10088035; doi:10.1021/acs.jproteome.2c00449)
Supplement: Supplementary file 4 — pr2c00449_si_004.pdf [file pr2c00449_si_004.pdf]

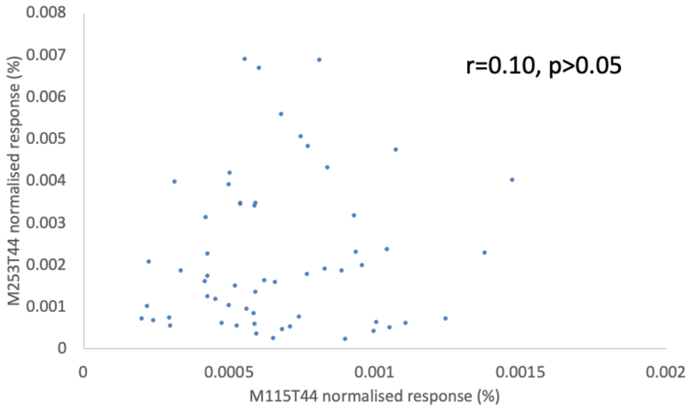

Supplementary File 31. Scatter plot visualising the normalised peaks areas (%) for two metabolite features (M115T44 and M253T44) to assess whether the two metabolite features are generated from the same or different metabolites. The Pearson correlation was $r=0.10$ and $p>0.05$. In metabolite annotation applied in this research, for two metabolite features to be reported as originating from the same metabolite then the criteria of $r>0.50$ and $p<0.05$ need to be fulfilled. Therefore the two metabolite features are reported as originating from two different metabolites.
